# Body-Mass Index and Pancreatic Cancer Incidence: A Pooled Analysis of Nine Population-Based Cohort Studies With More Than 340,000 Japanese Subjects

**DOI:** 10.2188/jea.JE20160193

**Published:** 2018-05-05

**Authors:** Yuriko N. Koyanagi, Keitaro Matsuo, Hidemi Ito, Akiko Tamakoshi, Yumi Sugawara, Akihisa Hidaka, Keiko Wada, Isao Oze, Yuri Kitamura, Rong Liu, Tetsuya Mizoue, Norie Sawada, Chisato Nagata, Kenji Wakai, Tomio Nakayama, Atsuko Sadakane, Keitaro Tanaka, Manami Inoue, Shoichiro Tsugane, Shizuka Sasazuki

**Affiliations:** 1 Department of Epidemiology, Nagoya University Graduate School of Medicine, Nagoya, Japan; 2 Division of Molecular and Clinical Epidemiology, Aichi Cancer Center Research Institute, Nagoya, Japan; 3 Division of Epidemiology and Prevention, Aichi Cancer Center Research Institute, Nagoya, Japan; 4 Department of Public Health, Hokkaido University Graduate School of Medicine, Sapporo, Japan; 5 Division of Epidemiology, Department of Public Health and Forensic Medicine, Tohoku University Graduate School of Medicine, Sendai, Japan; 6 Epidemiology and Prevention Division, Research Center for Cancer Prevention and Screening, National Cancer Center, Tokyo, Japan; 7 Department of Epidemiology and Preventive Medicine, Gifu University Graduate School of Medicine, Gifu, Japan; 8 Department of Social and Environmental Medicine, Osaka University Graduate School of Medicine, Osaka, Japan; 9 Department of Epidemiology and International Health, International Clinical Research Center, National Center for Global Health and Medicine, Tokyo, Japan; 10 Department of Preventive Medicine, Nagoya University Graduate School of Medicine, Nagoya, Japan; 11 Center for Cancer Control and Statistics, Osaka Medical Center for Cancer and Cardiovascular Diseases, Osaka, Japan; 12 Department of Epidemiology, Radiation Effects Research Foundation, Hiroshima, Japan; 13 Department of Preventive Medicine, Faculty of Medicine, Saga University, Saga, Japan; 14 AXA Department of Health and Human Security, Graduate School of Medicine, University of Tokyo, Tokyo, Japan

**Keywords:** pancreatic cancer, body mass index, cohort study, Japanese, pooled analysis

## Abstract

**Background:**

A high body mass index (BMI) has been proposed as an important risk factor for pancreatic cancer. However, this association of BMI with pancreatic cancer risk has not been confirmed in Asian populations.

**Methods:**

We evaluated the association between BMI (either at baseline or during early adulthood) and pancreatic cancer risk by conducting a pooled analysis of nine population-based prospective cohort studies in Japan with more than 340,000 subjects. Summary hazard ratios (HRs) were estimated by pooling study-specific HRs for unified BMI categories with a random-effects model.

**Results:**

Among Japanese men, being obese at baseline was associated with a higher risk of pancreatic cancer incidence (≥30 kg/m^2^ compared with 23 to <25 kg/m^2^, adjusted HR 1.71; 95% confidence interval [CI], 1.03–2.86). A J-shaped association between BMI during early adulthood and pancreatic cancer incidence was seen in men. In contrast, we observed no clear association among women, although there may be a positive linear association between BMI at baseline and the risk of pancreatic cancer (per 1 kg/m^2^, adjusted HR 1.02; 95% CI, 1.00–1.05).

**Conclusions:**

Pooling of data from cohort studies with a considerable number of Japanese subjects revealed a significant positive association between obesity and pancreatic cancer risk among men. This information indicates that strategies that effectively prevent obesity among men might lead to a reduced burden of pancreatic cancer, especially in Asian populations.

## INTRODUCTION

Pancreatic cancer is a major cause of cancer mortality in developed countries.^1^ In Japan, pancreatic cancer is the fourth-leading cause of cancer death, with 31,716 deaths occurring in 2014.^2^ This cancer has the highest case fatality of any major cancer, and 5-year survival rate among Japanese is only 7%.^3^ In recent years, the age-standardized incidence and mortality rates have increased significantly for both sexes in Japan.^4^ Since the early stages of pancreatic cancer do not usually produce symptoms, this cancer is generally diagnosed in the advanced stages. Moreover, an early detection method for pancreatic cancer has yet to be established. Efforts to reduce the burden of pancreatic cancer should therefore be strongly focused on primary prevention.

The World Cancer Research Fund (WCRF) and American Institute of Cancer Research (AICR) concluded that the positive association between body-mass index (BMI) and pancreatic cancer risk is convincing.^5^ Most of the findings on which this conclusion was came from Western populations, however, and it remains unclear whether it is also applicable to Asian populations, which substantially differ from Western populations in the prevalence of overweight and also in dietary factors, lifestyle characteristics, and genetic background. In fact, findings from prospective studies in Japan have been inconsistent,^6^^–^^9^ while two pooled analyses of pancreatic cancer mortality from Asian countries reported that there was no clear association.^10^^,^^11^

Several studies^6^^,^^8^^,^^12^^–^^15^ analyzing prospective data have examined the association between BMI during early adulthood (ages 18–21 years) and the risk of pancreatic cancer, with generally consistent evidence: all studies except one^8^ suggested a positive association between the two. The WCRF/AICR stated that greater childhood growth (marked as BMI at age ∼20 years) is a probable cause of pancreatic cancer.^5^ With respect to BMI change from younger age to older age, however, the results were not consistent.^5^^,^^12^^–^^15^ These findings are also mostly from Western populations, and evidence from Asian populations is again limited.

Evidence to date on the association between BMI and pancreatic cancer incidence from pooled analyses with substantial population sizes in Asia is scarce. In the present study, we evaluated the association between BMI and pancreatic cancer risk in Japan by conducting a pooled analysis of nine population-based prospective cohort studies comprising >340,000 subjects with unified BMI categories.

## METHODS

### Study population

Details of the study method have been provided elsewhere.^16^^–^^18^ In brief, the present study was conducted using data from nine representative ongoing large-scale population-based cohort studies in Japan, namely (i) the Japan Public Health Center-based Prospective Study (JPHC-I),^19^ (ii) the Japan Public Health Center-based Prospective Study (JPHC-II),^19^ (iii) the Japan Collaborative Cohort Study (JACC),^20^ (iv) the Miyagi Cohort Study (MIYAGI-I),^21^ (v) the Three-Prefecture Cohort Study in Miyagi (MIYAGI-II),^22^ (vi) the Three-Prefecture Cohort Study in Aichi (AICHI),^22^ (vii) the Takayama Study (TAKAYAMA),^8^ (viii) the Ohsaki Cohort Study (OHSAKI),^23^ and (ix) the Three-Prefecture Cohort Study in Osaka (OSAKA)^22^ (Table 1). All these studies started after the mid-1980s and enrolled >30,000 participants. Residence status in each study, including survival, was confirmed through the residential registry. Variables used in data linkage, censoring criteria, and the method to obtain information on cancer incidence in each study are provided in eTable 1. Each study was approved by its relevant institutional ethics review board.

**Table 1.** Characteristics of the cohort studies in the present pooled analysis

Study	Population	Age range at baseline, y	Year of baseline survey	Population size	Response rate (%) of the baseline questionnaire	Method of follow-up	For the present pooled analysis							

							Age range, y	Last follow-up time	Mean follow-up period, y	Outcome	Size of the cohort		Number of pancreatic cancer cases	
	
											Men	Women	Men	Women
JPHC-I														
	Japanese residents of 5 public health center areas in Japan	40–59	1990	61,595	82%	Cancer registry and death certificate	40–59	2010	18.6	Incidence	20,127	21,653	146	107
JPHC-II														
	Japanese residents of 6 public health center areas in Japan	40–69	1993–1994	78,825	80%	Cancer registry and death certificate	40–69	2010	15.6	Incidence	15,795	15,855	90	61
JACC														
	Residents from 45 areas throughout Japan	40–79	1988–1990	110,585	83%	Cancer registry (selected areas: 24) and death certificate	40–79	2009	12.9	Incidence	25,889	37,600	155	170
MIYAGI-I														
	Residents of 14 municipalities in Miyagi Prefecture, Japan	40–64	1990	47,605	92%	Cancer registry and death certificate	40–64	2003	15.7	Incidence	19,747	21,010	124	75
MIYAGI-II														
	Residents of 3 municipalities in Miyagi Prefecture, Japan	40+	1984	31,345	94%	Cancer registry and death certificate	40+	1992	7.7	Incidence	13,010	15,944	35	36
AICHI														
	Residents of 2 municipalities in Aichi Prefecture, Japan	40–103	1985	33,529	90%	Cancer registry and death certificate	40–103	2000	11.5	Incidence	15,738	17,777	79	59
OSAKA														
	Residents of 4 municipalities in Osaka Prefecture, Japan	40–97	1983–1985	35,755	84.51%	Cancer registry and death certificate	40–97	2000	12.3	Incidence	16,693	19,062	97	53
TAKAYAMA														
	Residents of Takayama city, Gifu Prefecture, Japan	35+	1992	31,552	85.30%	Cancer registry and death certificate	35–98	2008	13.8	Incidence	13,026	14,948	79	56
OHSAKI														
	Residents of 14 municipalities in Miyagi Prefecture, Japan	40–79	1994	52,029	95%	Cancer registry and death certificate	40–79	2003	9.0	Incidence	20,463	21,462	80	91

Total				482,820							**160,488**	**185,311**	**885**	**708**

AICHI, Aichi Cohort Study; JACC, The Japan Collaborative Cohort Study; JPHC, Japan Public Health Center-based Prospective Study; MIYAGI, The Miyagi Cohort Study; OHSAKI, Ohsaki Cohort Study.

Five studies (JPHC-I and -II, JACC, MIYAGI-II, and TAKAYAMA) have already published results on the association between BMI and the risk of pancreatic cancer in the respective cohort.^6^^–^^9^ In this study, we reanalyzed the results of each study using the updated dataset. Characteristics of the cohort studies in the present pooled analysis are shown in Table 1.

### Assessment of exposure

All studies collected information on height and weight at baseline using self-report. Five studies (JPHC-II, JACC, TAKAYAMA, MIYAGI-I, and OHSAKI) collected self-reported, recalled weight during early adulthood (age 20 or 21). Body mass index (BMI) was calculated as weight in kilograms divided by the square of the height in meters in their questionnaires. Several studies have reported the validity of self-reported height and weight; respective correlation coefficients for height and weight in both sexes were 0.93 and 0.97 in TAKAYAMA,^24^ 0.97 and 0.85 in MIYAGI-I,^25^ and 0.93 and 0.97 in OHSAKI.^26^ Correlation coefficients for comparisons of BMI estimated from the questionnaire with BMI from the actually measured weight and height were 0.89 in men and 0.90 in women in JPHC-I and -II,^27^ 0.91 for both sexes in MIYAGI-I,^25^ and 0.88 for both sexes in OHSAKI.^26^ JACC, which did not have information on the validation of BMI, utilized the same questions on weight and height as MIYAGI-I. MIYAGI-II, AICHI, and OSAKA also had no information on the validation of BMI, but used a similar question to that in JPHC-I and -II. Important covariates for pancreatic cancer, including smoking history, alcohol consumption, and diabetes, were also collected via a self-administered questionnaire.

### Assessment of outcome

We identified pancreatic cancer cases via local cancer registries or direct access to major local hospitals (eTable 1). In all studies except OSAKA, information on cancer diagnosis was collected for the whole population and was coded using the International Classification of Diseases for Oncology, Third Edition (ICD-O-3).^28^ In OSAKA, sites of any cancers were coded using the International Classification of Diseases, Ninth Revision (ICD-9),^29^ except for one city in Osaka where the ICD 10th Revision (ICD-10)^30^ was used. Study outcome was defined as the incidence of pancreatic cancer (ICD-9: 157.0–157.9, ICD-10 or ICD-O-3: C25.0–C25.9) during the follow-up period of each study. Participants were followed from the date of completion of the baseline questionnaire (JPHC-I and -II: 1990–1994, JACC: 1988–1990, MIYAGI-I: 1990, Miyagi-II: 1984, AICHI: 1985, OSAKA: 1983–1985, TAKAYAMA: 1992, and OHSAKI: 1994) until the last date of follow-up in each study (JPHC-I and -II: 2010, JACC: 2009, MIYAGI-I: 2003, MIYAGI-II: 1992, AICHI: 2000, OSAKA: 2000, TAKAYAMA: 2008, and OHSAKI: 2003), date of death, date of loss to follow-up, or date of diagnosis of pancreatic cancer, whichever occurred first.

### Exclusions

Participants were excluded from all analyses if they had missing or extreme values of BMI (BMI <14 or >40 kg/m^2^) or extreme values of BMI change (more than 3 standard deviations from the mean). Patients with cancer at baseline were also excluded from all analyses.

### Statistical analysis

For categorical analyses, BMI (kg/m^2^) at baseline was divided by sex-specific categories using identical cut points for men at <19, 19 to <21, 21 to <23, 23 to <25, 25 to <27, 27 to <30, and ≥30; and for women at <19, 19 to <21, 21 to <23, 23 to <25, 25 to <27, and ≥27. We defined 23 to <25 kg/m^2^ as the reference category. BMI (kg/m^2^) during early adulthood was divided using the same categories as BMI at baseline. BMI change (BMI at baseline–BMI during early adulthood) was classified using the following cut points for men: <−4, −4 to <−2, −2 to <2, 2 to <5, ≥5; and for women: <−2, −2 to <2, 2 to <5, 5 to <8, and ≥8. We defined −2 to <2 kg/m^2^ as the reference category. Smoking history was evaluated as pack-years, calculated by multiplying the number of packs consumed per day by the number of years of smoking, and then classified into three categories: 0, 0< and ≤20, and >20. Alcohol consumption for men was categorized into nondrinker, occasional drinker (less than once a week), and current drinker (<23, 23 to <46, 46 to <69, and ≥69 ethanol g/day), and that for women as nondrinker, occasional drinker and current drinker (<23 and ≥23 ethanol g/day). Current drinkers were further categorized by the amount of alcohol consumed using cut points per 23 g ethanol, on the basis that some questionnaires collected data on the amount consumed in terms of one “go” (180 mL) of Japanese sake equivalent, which contains 23 g ethanol. A history of diabetes was categorized as “Yes” or “No”. Missing data of smoking history, alcohol consumption, and diabetes were coded using dummy variables.

To be pooled in the estimation of summary statistics, we conducted study-specific analyses by sex.^16^ Each analysis used a Cox proportional hazards model to estimate the hazard ratios (HR) and their two-sided 95% confidence intervals (CI) of pancreatic cancer by each BMI category. We assessed the assumption of proportional hazards graphically using log-negative-log plot, and detected no major violations of the proportional hazards assumption. All studies analyzed two models in the estimation of HRs: model 1, age (and area within each study for JPHC and JACC)-adjusted HRs; and model 2, smoking history, alcohol consumption, and diabetes in addition to model 1. Model 2 of BMI change was additionally adjusted for BMI during early adulthood (continuous values). We also evaluated HRs by excluding cases diagnosed early within three years after enrollment in each study. All analyses were performed by SAS version 9.3 (SAS Institute, Inc., Cary, NC, USA) or STATA version 14.1 (Stata Corporation, College Station, TX, USA).

We applied a random-effects model^31^ to obtain a single pooled estimate of the HR from the individual studies for each category.^16^ The extent of heterogeneity for each category was indicated by Cochran’s *Q*-statistic, which was considered statistically significant when *P* < 0.10. The *I*^2^-statistic was also reported to describe the percentage of total variation in the study-specific HRs which was due to heterogeneity.^32^ The dose-response relationship was examined using models in which actual BMI values were included as an explanatory variable, which would provide HRs by a 1-kg/m^2^ increase in BMI. Meta-analyses were done using the Stata’s ‘metan’ command (http://www.stata.com/stb/stb44).

We also evaluated the joint effect of a combination of BMI at baseline and BMI during early adulthood. For this analysis, BMI at baseline and BMI during early adulthood were coded as <21, 21 to <25, and ≥25 kg/m^2^; accordingly, nine combinations were evaluated by setting BMI at baseline and BMI during early adulthood, with 21 to 25 as the reference category for both. This combined analysis was done using model 2 to estimate HRs. An interaction between BMI at baseline and BMI during early adulthood was assessed by adding a cross-product term (coding the two variables as ordinal variables: 0, 1, 2) in the model.

## RESULTS

The present pooled analysis included nine cohort studies comprising 345,799 subjects (160,488 males and 185,311 females) with 1,593 incident pancreatic cancer cases (885 males and 708 females) during 4,361,312 person-years of follow-up (average follow-up: 13.0 years) (Table 1). At baseline, those with a BMI ≥25 kg/m^2^ accounted for 22.9% of males and 24.9% of females. These values are comparable to those reported in national surveys of the same age group.^33^

### Males

Table 2, Table 3, eTable 2, eTable 3, and eTable 4 show results for males. As shown in Table 2, a statistically significant increase in risk of pancreatic cancer was observed for the highest category of BMI at baseline (≥30 kg/m^2^ compared with 23 to <25 kg/m^2^, adjusted [model 2] HR [aHR] 1.71; 95% CI, 1.03–2.86). Although HRs with the exclusion of early cases were not significant for those with a BMI at baseline ≥30, trends of association were consistent (aHR 1.65; 95% CI, 0.94–2.88). Those with a BMI at baseline <21 kg/m^2^ appeared to be at slightly increased risk of pancreatic cancer, although without statistical significance. For BMI in early adulthood, we observed a J-shaped association with pancreatic cancer risk (BMI <19: aHR 1.13; 95% CI, 0.74–1.72; BMI 27 to <30: aHR 1.46; 95% CI, 0.92–2.34; and BMI ≥30: aHR 2.32; 95% CI, 0.84–6.40) (Table 3).

**Table 2.** Hazard ratios (HR) and 95% confidence intervals (CI) for pancreatic cancer risk according to category of BMI at baseline among males

	Categories of BMI at baseline (kg/m^2^)							Trend (per 1 kg/m^2^)	Trend *P*	Heterogeneity [*P*, *I*^2^ (%)]	
	
	<19	19 to <21	21 to <23	23 to <25	25 to <27	27 to <30	≥30			For trend	For the highest category
Number of subjects (*N*)	10,043	27,472	42,006	39,963	22,006	10,754	2,672				
Person-years (*N*)	111,259.9	345,620.1	544,890.7	532,025.8	295,509.3	145,176.7	34,168.7				
Number of cases (*N*)	70	183	234	199	95	50	18				
Crude rate (per 100,000)	62.9157	52.9483	42.9444	37.4042	32.1479	34.4408	52.6798				
HR (model 1) overall	1.24 (0.85–1.81)	1.26 (0.98–1.63)	1.08 (0.90–1.31)	Reference	0.91 (0.71–1.17)	1.11 (0.81–1.53)	**1.73 (1.04–2.87)**	0.97 (0.94–1.01)	0.112	*P* = 0.065, 45.7	*P* = 0.890, 0
HR (model 2) overall	1.22 (0.85–1.77)	1.26 (0.98–1.63)	1.08 (0.89–1.31)	Reference	0.91 (0.71–1.17)	1.12 (0.82–1.54)	**1.71 (1.03–2.86)**	0.97 (0.94–1.01)	0.113	*P* = 0.094, 41.0	*P* = 0.931, 0
HR (model 1) excluding early cases	1.14 (0.73–1.79)	1.22 (0.92–1.62)	1.00 (0.81–1.22)	Reference	0.87 (0.66–1.16)	1.00 (0.69–1.45)	1.66 (0.96–2.89)	0.97 (0.93–1.01)	0.106	*P* = 0.075, 44.0	*P* = 0.908, 0
HR (model 2) excluding early cases	1.14 (0.73–1.78)	1.22 (0.92–1.62)	1.00 (0.82–1.23)	Reference	0.87 (0.65–1.17)	1.01 (0.71–1.45)	1.65 (0.94–2.88)	0.97 (0.93–1.01)	0.090	*P* = 0.122, 37.1	*P* = 0.941, 0

BMI, body mass index.

Model 1 adjusted for age and area (within each study for JPHC and JACC). Model 2 adjusted for age, area, smoking (pack-years [PY] = 0, 0 < PY ≤ 20, >20, or unknown), drinking (never, <1 week/day, current <23 g/day, 23 to <46 g/day, 46 to <69 g/day, ≥69 g/day, or unknown), and history of diabetes (yes, no, or unknown). HRs values in bold show statistical significance.

**Table 3.** Hazard ratios (HR) and 95% confidence intervals (CI) for pancreatic cancer risk according to category of BMI during early adulthood among males

	Categories of BMI during early adulthood (kg/m^2^)							Trend (per 1 kg/m^2^)	Trend *P*	Heterogeneity [*P*, *I*^2^ (%)]	
	
	<19	19 to <21	21 to <23	23 to <25	25 to <27	27 to <30	≥30			For trend	For the highest category
Number of subjects (*N*)	7,339	24,334	30,249	18,606	6,362	2,649	584				
Person-years (*N*)	97,321.3	328,474.9	402,153.8	236,802.9	77,447.3	31,163.0	6,738.9				
Number of cases (*N*)	36	107	166	119	47	22	4				
Crude rate (per 100,000)	36.99	32.57	41.28	50.25	60.69	70.60	59.36				
HR (model 1) overall	1.05 (0.70–1.60)	0.87 (0.67–1.14)	0.95 (0.75–1.21)	Reference	1.08 (0.76–1.51)	1.44 (0.91–2.30)	2.06 (0.75–5.66)	1.02 (0.99–1.06)	0.232	*P* = 0.740, 0	*P* = 0.997, 0
HR (model 2) overall	1.13 (0.74–1.72)	0.90 (0.69–1.19)	0.99 (0.77–1.25)	Reference	1.10 (0.78–1.56)	1.46 (0.92–2.34)	2.32 (0.84–6.40)	1.02 (0.98–1.06)	0.340	*P* = 0.791, 0	*P* = 0.922, 0
HR (model 1) excluding early cases	1.13 (0.69–1.86)	0.86 (0.64–1.16)	0.98 (0.73–1.30)	Reference	1.08 (0.74–1.57)	1.64 (0.98–2.73)	2.58 (0.94–7.13)	1.03 (0.99–1.07)	0.188	*P* = 0.541, 0	*P* = 0.966, 0
HR (model 2) excluding early cases	1.20 (0.72–1.99)	0.89 (0.66–1.21)	0.99 (0.75–1.32)	Reference	1.09 (0.75–1.60)	1.64 (1.00–2.70)	**2.81 (1.01–7.80)**	1.02 (0.98–1.06)	0.304	*P* = 0.603, 0	*P* = 0.938, 0

Model 1 adjusted for age and area (within each study for JPHC and JACC). Model 2 adjusted for age, area, smoking (pack-years [PY] = 0, 0 < PY ≤ 20, >20, or unknown), drinking (never, <1 week/day, current <23 g/day, 23 to <46 g/day, 46 to <69 g/day, ≥69 g/day, or unknown), and history of diabetes (yes, no, or unknown). HRs values in bold show statistical significance. BMI during early adulthood was evaluated only in JPHC2, JACC, Takayama, Miyazaki, and Ohsaki.

To assess the effect modification of smoking status, we stratified male participants into two subgroups (never smoking group and ever smoking group), and estimated HR of pancreatic cancer by each category of BMI at baseline. After adjusting for age, area (within each study for JPHC and JACC), alcohol consumption, and history of diabetes, we observed similar tendency as the analysis without stratification (Table 2) in the two subgroups (eTable 2). Specifically, a significant increase in risk of pancreatic cancer was observed for the highest category of BMI at baseline in both subgroups. In addition, there was a consistent risk elevation in those with lower BMI across never smokers and ever smokers although without statistical significance in never smoking group. However, the result should be interpreted with caution because stratification attenuates the statistical power especially in never smoking group.

A statistically significantly decrease in risk of pancreatic cancer among men was observed in the highest category of BMI change (≥5 kg/m^2^: aHR 0.55; 95% CI, 0.32–0.96) (eTable 3). Results remained similar after model 2 was additionally adjusted for BMI during early adulthood. In addition, when we evaluated a combination of BMI at baseline and BMI during early adulthood (eTable 4), we observed a similar tendency as for BMI change, suggesting that BMI gain might be associated with a lower risk of pancreatic cancer (*P* for interaction = 0.230). To assess the association between pancreatic cancer and BMI gain, we further evaluated the distribution of BMI during early adulthood in each category of BMI change (eTable 5 and eTable 6). As shown, many of those with a BMI <19 kg/m^2^ during early adulthood were categorized in the highest category of BMI change. The prevalence of a BMI <19 during early adulthood was 8.1% in all subjects but 22.0% in those in the highest category of BMI change.

### Females

Table 4, Table 5, eTable 7, and eTable 8 show results for females. Lower BMI at baseline appears to have been associated with lower risk of pancreatic cancer, but the results were not significant (Table 4). Trend in BMI at baseline may suggest that higher BMI at baseline was associated with weakly increased pancreatic cancer risk (per 1 kg/m^2^, aHR 1.02; 95% CI, 1.00–1.05), although the *P* value was only marginally statistically significant (Table 4). On the other hand, we observed no clear association between BMI during early adulthood and the risk of pancreatic cancer (Table 5). BMI gain might be associated with higher risk of pancreatic cancer (eTable 7), but a similar tendency was not observed in evaluation of the joint effects of BMI at baseline and BMI during early adulthood (*P* for interaction = 0.333) (eTable 8).

**Table 4.** Hazard ratios (HR) and 95% confidence intervals (CI) for pancreatic cancer risk according to category of BMI at baseline among females

	Categories of BMI at baseline (kg/m^2^)						Trend (per 1 kg/m^2^)	Trend *P*	Heterogeneity [*P*, *I*^2^ (%)]	
	
	<19	19 to <21	21 to <23	23 to <25	25 to <27	≥27			For trend	For the highest category
Number of subjects (*N*)	14,737	31,606	45,779	39,842	24,086	19,710				
Person-years (*N*)	177,760.5	416,439.0	620,291.6	544,977.2	328,792.9	264,400.0				
Number of cases (*N*)	47	112	150	167	85	93				
Crude rate (per 100,000)	26.44	26.89	24.18	30.64	25.85	35.17				
HR (model 1) overall	0.71 (0.49–1.03)	0.89 (0.68–1.18)	**0.79 (0.62–0.99)**	Reference	0.82 (0.60–1.12)	1.06 (0.82–1.37)	1.02 (1.00–1.05)	0.072	*P* = 0.627, 0	*P* = 0.673, 0
HR (model 2) overall	0.69 (0.48–1.00)	0.90 (0.68–1.18)	0.80 (0.64–1.00)	Reference	0.84 (0.61–1.15)	1.04 (0.80–1.35)	1.02 (1.00–1.05)	0.086	*P* = 0.655, 0	*P* = 0.706, 0
HR (model 1) excluding early cases	0.76 (0.50–1.15)	0.92 (0.70–1.21)	0.82 (0.62–1.09)	Reference	0.84 (0.61–1.16)	1.13 (0.85–1.49)	1.03 (1.00–1.05)	0.072	*P* = 0.550, 0	*P* = 0.438, 0
HR (model 2) excluding early cases	0.73 (0.48–1.12)	0.93 (0.71–1.21)	0.83 (0.63–1.08)	Reference	0.87 (0.64–1.19)	1.11 (0.84–1.47)	1.02 (1.00–1.05)	0.082	*P* = 0.556, 0	*P* = 0.459, 0

BMI, body mass index.

Model 1 adjusted for age and area (within each study for JPHC and JACC). Model 2 adjusted for age, area, smoking (pack-years [PY] = 0, 0 < PY ≤ 20, >20, or unknown), drinking (never, <1 week/day, current <23 g/day, ≥23 g/day, or unknown), and history of diabetes (yes, no, or unknown). HRs values in bold show statistical significance.

**Table 5.** Hazard ratios (HR) and 95% confidence intervals (CI) for pancreatic cancer risk according to category of BMI during early adulthood among females

	Categories of BMI during early adulthood (kg/m^2^)							Trend *P*	Heterogeneity [*P*, *I*^2^ (%)]	
	
	<19	19 to <21	21 to <23	23 to <25	25 to <27	≥27	Trend (per 1 kg/m^2^)		For trend	For the highest category
Number of subjects (*N*)	14,139	28,651	30,660	17,693	7,575	3,698				
Person-years (*N*)	191,598.8	393,283.2	416,524.7	236,854.3	97,697.5	45,357.3				
Number of cases (*N*)	38	99	132	79	36	15				
Crude rate (per 100,000)	19.83	25.17	31.69	33.35	36.85	33.07				
HR (model 1) overall	0.82 (0.55–1.22)	1.00 (0.67–1.50)	1.06 (0.80–1.41)	Reference	0.95 (0.54–1.66)	0.75 (0.42–1.35)	0.99 (0.95–1.04)	0.754	*P* = 0.208, 32.0	*P* = 0.381, 4.4
HR (model 2) overall	0.85 (0.56–1.27)	1.03 (0.70–1.51)	1.13 (0.84–1.50)	Reference	1.01 (0.64–1.59)	0.77 (0.44–1.36)	0.99 (0.95–1.04)	0.744	*P* = 0.252, 25.4	*P* = 0.586, 0
HR (model 1) excluding early cases	0.95 (0.62–1.44)	1.06 (0.67–1.69)	1.16 (0.86–1.58)	Reference	1.00 (0.60–1.67)	0.87 (0.48–1.57)	0.99 (0.94–1.04)	0.709	*P* = 0.194, 34.1	*P* = 0.405, 0.1
HR (model 2) excluding early cases	0.99 (0.64–1.52)	1.09 (0.70–1.69)	1.25 (0.91–1.71)	Reference	1.07 (0.69–1.67)	0.90 (0.50–1.64)	0.99 (0.95–1.04)	0.715	*P* = 0.232, 28.4	*P* = 0.605, 0

Model 1 adjusted for age and area (within each study for JPHC and JACC). Model 2 adjusted for age, area, smoking (pack-years [PY] = 0, 0 < PY ≤ 20, >20, or unknown), drinking (never, <1 week/day, current <23 g/day, ≥23 g/day, or unknown), and history of diabetes (yes, no, or unknown). HRs values in bold show statistical significance. BMI during early adulthood was evaluated only in JPHC II, JACC, Takayama, Miyazaki, and Ohsaki.

## DISCUSSION

This large study, the first pooled analysis to examine an association between BMI and pancreatic cancer incidence in the Japanese population, was conducted as a pooled analysis of data from nine population-based cohort studies comprising >340,000 subjects. Obesity at baseline was associated with a statistically significantly higher risk of pancreatic cancer incidence among Japanese men. For BMI during early adulthood, we observed a J-shaped association with pancreatic cancer incidence in men. Among Japanese women, in contrast, results suggested a positive linear association between BMI at baseline and pancreatic cancer incidence.

Our study did not support the internationally reported association between obesity and pancreatic cancer among women.^13^^–^^15^ This may be partly because of the small number of women in the higher BMI categories and the fact that we did not define the category that had the smallest risk of pancreatic cancer as the reference category. With respect to men, in contrast, a statistically significant increase in risk of pancreatic cancer was observed for the highest category of BMI at baseline (≥30 kg/m^2^), consistent with previous large studies from Western countries.^12^^–^^15^ In two pooled analyses of Asian populations that reported no clear association between BMI and pancreatic cancer risk,^10^^,^^11^ mortality was used as an end point. Using mortality instead of incidence is reasonable because pancreatic cancer has high case fatality and short survival time. On the other hand, early detection of pancreatic cancer is difficult, and diagnosis of pancreatic cancer requires advanced level of medical standards. Therefore, the two studies might include pancreatic cancer death without being diagnosed, which resulted in the possibility of misclassification for pancreatic cancer mortality. We consider that the difference of diagnostic accuracy may contribute to the discrepant finding. To date, only a few prospective studies have evaluated the association between pancreatic cancer and BMI at baseline in Japan.^6^^–^^9^ Three studies^6^^,^^8^^,^^9^ showed no significant association, while the fourth^7^ showed an inverse association among men and no association among women. The reason for this heterogeneity might be partly due to the limited statistical power of each study to detect the association. Our present pooled approach has likely resolved the statistical limitations of each of the individual studies, and the stability of the resulting estimation is therefore of marked importance to this field.

Evidence to date suggests that BMI at baseline has a positive linear association with pancreatic cancer risk. A meta-analysis using studies mainly from Western countries^34^ and three pooled analyses from Western countries^13^^,^^14^^,^^35^ showed a significant positive linear association, whereas a pooled analysis from Asian countries^10^ showed a non-significant positive linear association (per 5 kg/m^2^, RR 1.02; 95% CI, 0.83–1.25). In the present study, the aHR of BMI at baseline (per 1 kg/m^2^) was 0.97 (95% CI, 0.94–1.01) in men and 1.02 (95% CI, 1.00–1.05) in women (Table 2 and Table 4). Unlike these former pooled analyses, we did not observe a positive linear association among men, owing to the point estimates being above unity for those with a BMI at baseline <21 kg/m^2^.

Although our study is the first pooled analysis of the association between BMI in early adulthood and pancreatic cancer incidence in Asia, two prospective studies have also evaluated this association in Japan: the first^6^ showed a significant association between obesity in early adulthood and pancreatic cancer death in men, while the second^8^ showed no association. In the present study, we found that being either overweight (BMI ≥25 to <30 kg/m^2^) or obese (BMI ≥30 kg/m^2^) during early adulthood appeared to be associated with a higher risk of pancreatic cancer in men. In addition, men with a BMI <25 kg/m^2^ during early adulthood appeared to be at lower risk, even if they gained weight after early adulthood (Table 3 and eTable 4). These findings suggest that the proper management of body weight up to age 20 years of age may be important for pancreatic cancer prevention.

Among other findings, a BMI gain ≥5 kg/m^2^ was associated with lower pancreatic cancer incidence among men. There might be several explanations for this association. First, as shown in eTable 5, many of those with a BMI <19 kg/m^2^ during early adulthood were categorized in the highest category of BMI change. It is possible that those with a BMI <19 kg/m^2^ during early adulthood were neither overweight nor obese at baseline even if they had gained weight more than 5 kg/m^2^ after early adulthood, which partly contributed to an unexpected reduction in pancreatic cancer incidence. Another explanation is that the inverse association between pancreatic cancer risk and BMI gain might be confounded by smoking due to lower BMI gain among smokers. In fact, one pooled analysis reported that BMI gain, measured as a continuous variable, was significantly associated with lower pancreatic cancer mortality for current smokers, although it was associated with weakly increased pancreatic cancer mortality among overall subjects.^13^ Nevertheless, this reduced risk by BMI gain might be due to chance because of limited number of events (*n* = 15) in this large pooled analysis.

Over the last several decades, average BMI in the Japanese population has increased constantly.^33^^,^^36^ In addition, the most recent survey in Japan^37^ showed an increasing trend of overweight and obesity for male adults in all age groups, although the prevalence of obesity still remains low compared with Western populations. Our finding of a concrete association between pancreatic cancer risk and BMI suggests that the increased prevalence of overweight and obesity among Japanese men over decades might partly explain the recent increases in pancreatic cancer incidence in Japan.^4^

On the other hand, the prevalence of diabetes has rapidly increased in recent decades,^38^^,^^39^ which might contribute to the increase in pancreatic cancer incidence.^40^ In the present study, the significant positive association persisted when adjusted by history of diabetes, which indicated that obesity at baseline was associated with a higher risk of pancreatic cancer incidence that is independent of diabetes. However, since BMI partly could increase the risk of pancreatic cancer due to its effect on diabetes, diabetes may also be regarded as an intermediate variable.^34^ Indeed, it is difficult to exclude the effect of diabetes completely from the analysis. Therefore, this point should be investigated in future studies.

The present study has several strengths. It included most of the ongoing, large-scale prospective cohorts in Japan, with overlapping birth generations of the study subjects. The total number of subjects in this analysis is very large, warranting sufficient statistical power to detect an association between BMI and pancreatic cancer risk. Therefore, pooling of these cohorts allows for stable summary quantitative estimates of the effect of BMI among Japanese. Using incidence instead of mortality as an end point is advantageous because it enables the direct assessment of the risk contribution of BMI. In addition, because this study was not based on a meta-analysis of published studies, the possibility of publication bias is small.^18^ BMI was measured before pancreatic cancer incidence in all studies included in this pooled analysis, which excludes the possibility of selection bias and recall bias. In addition, the categories for BMI and the covariates used were identical across studies, which removed a potential source of heterogeneity that can occur when conducting a meta-analysis of published studies.^17^ Lastly, we observed non-significant heterogeneity of association across the studies.

Several potential limitations of the present study warrant mention. First, all studies collected information on height and weight using self-report. Previous research^41^ has shown that self-reported past body weight is highly correlated with measured weight, but that the accuracy of recall is influenced by age or elapsed time, current body size, and body weight variability. Therefore, although errors in self-reported information are unlikely to be strongly associated with the risk of pancreatic cancer incidence, the possibility remains of reporting bias, recall bias, and differential misclassification of BMI during early adulthood. With regard to BMI at baseline, most of the studies used validated questionnaires^24^^–^^27^ or equivalent questionnaires for current BMI measurement, and any impact of error in this analysis appears limited, if present at all. Second, we were unable to consider changes in BMI and potential confounders over time after enrollment because our analyses were conducted using only a baseline questionnaire. Third, although we considered potential confounders in the analysis, the possibility of residual confounding can never be fully excluded. Fourth, there is a possibility that the healthy participants with adequate BMI tend to move to the other regions for some reasons and are more likely lost to follow-up compared with the obese participants. This potential selection bias might have caused the underestimation of HRs for the participants with adequate BMI. Therefore, the result of the present study should be interpreted carefully. Lastly, as this is the aggregation of cohort studies, other anthropometric measurements, such as waist circumference or waist-to-hip ratio, were not evaluated because not all the studies collected such data. Epidemiological findings, mostly from Western populations, showed that alternative measures of adiposity might also be important indicators of pancreatic cancer risk.^13^^,^^34^^,^^42^ To promote understanding of the association between anthropometric factors and pancreatic cancer risk in Asian population, evaluation of comprehensive anthropometric measurements are needed in future studies.

In conclusion, this study revealed a possible association between BMI (either at baseline or during early adulthood) and pancreatic cancer incidence in an Asian population. Pooling of data from cohort studies with a considerable number of Japanese subjects revealed a significant positive association between obesity and pancreatic cancer risk among men. In contrast, we observed no clear association among women, although we did see a tendency toward a positive linear association between BMI at baseline and the risk of pancreatic cancer. This information indicates that reducing the burden of pancreatic cancer will require effective strategies to prevent obesity, particularly in Asian males.

## APPENDIX A. SUPPLEMENTARY DATA

eTable 1. Details of the study method in each study

eTable 2. Hazard ratios (HR) and 95% confidence intervals (CI) for pancreatic cancer risk according to category of BMI at baseline stratified by smoking status among males

eTable 3. Hazard ratios (HR) and 95% confidence intervals (CI) for pancreatic cancer risk according to category of BMI change among males

eTable 4. Hazard ratios (HR) and 95% confidence intervals (CI) for pancreatic cancer risk according to BMI during early adulthood stratified by BMI at baseline among males

eTable 5. Distribution of BMI during early adulthood in each category of BMI change among males

eTable 6. Distribution of BMI during early adulthood in each category of BMI change among males

eTable 7. Hazard ratios (HR) and 95% confidence intervals (CI) for pancreatic cancer risk according to category of BMI change among females

eTable 8. Hazard ratios (HR) and 95% confidence intervals (CI) for pancreatic cancer risk according to BMI during early adulthood stratified by BMI at baseline among females
