# Supplementary material for: Body-Mass Index and Pancreatic Cancer Incidence: A Pooled Analysis of Nine Population-Based Cohort Studies With More Than 340,000 Japanese Subjects
Source: J Epidemiol. 2018 May 5;28(5):245–52. doi: 10.2188/jea.JE20160193 (PMC5911675; doi:10.2188/jea.JE20160193)
Supplement: Supplementary file 1 [file je-28-245-s001.pdf]

eTable 1. Details of the study method in each study

Study	Variables used in data linkage	Censoring date	Methods to obtain information on cancer incidence
JPHC-I	First and last names, sex, date of birth, and address	Date of death, date of emigration, or date of last follow-up, whichever comes first.	Local cancer registries or direct access to major local hospitals
JPHC-II	First and last names, sex, date of birth, and address	Date of death, date of emigration, or date of last follow-up, whichever comes first.	Local cancer registries or direct access to major local hospitals
JACC	First and last names, sex, date of birth, and address	Date of death or date of emigration	Local cancer registries or direct access to major local hospitals
MIYAGI-I	First and last names, sex, date of birth, date of death, and address	Date of death or date of emigration	Local cancer registry
MIYAGI-II	First and last names, sex, date of birth, date of death, and address	Date of death or date of emigration	Local cancer registry
AICHI	First and last names, sex, date of birth, and address	Date of emigration	Local cancer registry
OSAKA	First and last names, sex, date of birth, and address	Date of death, date of emigration, or date of the end of the study period, whichever comes first.	Local cancer registries
TAKAYAMA	First and last names, date of birth, and address	Date of death or date of emigration	Local cancer registries or direct access to major local hospitals
OHSAKI	First and last names, sex, date of birth, and address	Date of death or date of emigration	Local cancer registry

AICHI, Aichi Cohort Study; JACC, The Japan Collaborative Cohort Study; JPHC, Japan Public Health Center-based prospective Study; MIYAGI, The Miyagi Cohort Study; OHSAKI, Ohsaki Cohort Study.

eTable 2. Hazard ratios (HR) and 95% confidence intervals (CI) for pancreatic cancer risk according to category of BMI at baseline stratified by smoking status among males

Smoking status		Categories of BMI at baseline (kg/m^2)						
		**<19**	**19 to <21**	**21 to <23**	**23 to <25**	**25 to < 27**	**27 to <30**	**≥30**
**Never**	**Number of subjects (*N*)**	1,384	4,177	7,299	8,081	4,963	2,569	620
	**Person-years (*N*)**	16,272.4	55,162.6	99,948.9	112,181.3	70,099.5	35,966.0	8,288.7
	**Number of cases (*N*)**	6	19	35	27	14	14	2
	**Crude rate (per 100,000)**	36.87	34.44	35.02	24.07	19.97	38.93	24.13
	**HR overall**	1.58 (0.54–4.61)	1.23 (0.67–2.27)	1.37 (0.81–2.30)	Reference	1.28 (0.62–2.67)	1.67 (0.82–3.38)	**11.21 (1.87–67.35)**
**Ever**	**Number of subjects (*N*)**	7,122	19,837	30,242	28,103	15,186	7,207	1,795
	**Person-years (*N*)**	79,282.6	251,781.6	393,602.8	375,495.1	204,026.5	98,053.7	23,170.1
	**Number of cases (*N*)**	48	144	171	148	69	34	15
	**Crude rate (per 100,000)**	60.54	57.19	43.44	39.41	33.82	34.67	64.74
	**HR overall**	1.31 (0.84–2.05)	**1.35 (1.07–1.70)**	1.05 (0.84–1.31)	Reference	0.92 (0.69–1.23)	1.05 (0.72–1.54)	**2.02 (1.14–3.58)**

BMI, body mass index.

HRs adjusted for age, area (within each study for JPHC and JACC), drinking (never, <1 week/day, current <23 g/day, 23 to <46 g/day, 46 to <69 g/day, ≥69 g/day, or unknown), and history of diabetes (yes, no, or unknown). HRs values in bold show statistical significance.

eTable 3. Hazard ratios (HR) and 95% confidence intervals (CI) for pancreatic cancer risk according to category of BMI change among males

	**Categories of BMI change (BMI at baseline - BMI during early adulthood, (kg/m^2))**					**Trend (per 1 kg/m^2)**	**Trend *P***	**Heterogeneity [*P*, I^2 (%)]**	
	**<-4**	**-4 to <-2**	**-2 to <2**	**2 to <5**	**≥5**			**For trend**	**For the highest category**
**Number of subjects (*N*)**	8,467	13,181	44,052	18,799	5,624				
**Person-years (*N*)**	102,871.1	166,715.5	583,544.9	250,563.5	76,406.9				
**Number of cases (*N*)**	41	88	254	103	15				
**Crude rate (per 100,000)**	39.86	52.78	43.53	41.11	19.63				
**HR (model 1) overall**	0.91 (0.65–1.27)	1.11 (0.86–1.42)	Reference	0.97 (0.69–1.36)	**0.54 (0.31–0.94)**	0.98 (0.92–1.04)	0.407	*P* = 0.005, 73.2	*P* = 0.356, 7.4
**HR (model 2) overall**	0.89 (0.63–1.25)	1.05 (0.82–1.35)	Reference	0.96 (0.69–1.33)	**0.55 (0.32–0.96)**	0.98 (0.92–1.04)	0.444	*P* = 0.010, 69.8	*P* = 0.363, 6.0
**HR (model 2)† overall**	0.98 (0.68–1.40)	1.09 (0.85–1.41)	Reference	0.90 (0.62–1.30)	**0.46 (0.27–0.79)**	0.95 (0.90–1.01)	0.112	*P* = 0.045, 58.9	*P* = 0.982, 0
**HR (model 1) excluding early cases**	0.85 (0.59–1.24)	1.10 (0.85–1.44)	Reference	0.96 (0.68–1.34)	0.56 (0.26–1.17)	0.98 (0.92–1.04)	0.484	*P* =0.005, 73.1	*P* = 0.171, 40.2
**HR (model 2) excluding early cases**	0.86 (0.59–1.26)	1.03 (0.79–1.36)	Reference	0.94 (0.69–1.29)	0.57 (0.27–1.19)	0.98 (0.92–1.04)	0.501	*P* = 0.010, 69.7	*P* = 0.172, 39.9
**HR (model 2)† excluding early cases**	0.92 (0.62–1.37)	1.02 (0.78–1.35)	Reference	0.94 (0.67–1.30)	**0.47 (0.26–0.84)**	0.96 (0.90–1.03)	0.235	*P* =0.025, 64.0	*P* =0.943, 0

BMI, body mass index.

Model 1 adjusted for age and area (within each study for JPHC and JACC). Model 2 adjusted for age, area, smoking (pack-years[PY]=0, 0<PY≤20, >20, or unknown), drinking (never, <1 week/day, current <23 g/day, 23 to <46 g/day, 46 to <69 g/day, ≥69 g/day, or unknown), and history of diabetes (yes, no, or unknown). HRs values in bold show statistical significance. BMI during early adulthood was evaluated only in JPHC2, JACC, Takayama, Miyazaki, and Ohsaki.

† HRs were additionally adjusted for BMI during early adulthood (continuous).

eTable 4. Hazard ratios (HR) and 95% confidence intervals (CI) for pancreatic cancer risk according to BMI during early adulthood stratified by BMI at baseline among males

	**BMI at baseline (kg/m²)**					
	**<21**		**21 to <25**		**≥25**	
**BMI during early adulthood (kg/m²)**	**Cases / Subjects (Person-years)**	**HR (95% CI) [HR (95%CI]†**	**Cases / Subjects (Person-years)**	**HR (95% CI) [HR (95%CI]†**	**Cases / Subjects (Person-years)**	**HR (95% CI) [HR (95%CI]†**
**<21**	75 / 11,331 (148,527.65)	**1.38 (1.00–1.90)** [1.39 (0.92–2.11)]	58 /16,155 (220,156.84)	0.73 (0.49–1.09) [0.67 (0.39–1.15)]	10 / 4,108 (56,404.66)	0.55 (0.27–1.12) [0.61 (0.29–1.27)]
**21 to <25**	69 / 8,620 (102,197.87)	1.09 (0.79–1.52) [1.08 (0.69–1.68)]	160 / 27,092 (358,664.09)	Reference	56 /13,112 (177,788.43)	0.84 (0.59–1.19) [0.79 (0.52–1.18)]
**≥25**	4 /674 (6,680.21)	1.83 (0.67–5.03) [2.36 (0.85–6.51)]	47 / 4,940 (58,068.39)	1.32 (0.87–2.02) [1.31 (0.86–1.98)]	22 / 4,008 (50,775.56)	1.00 (0.64–1.56) [1.09 (0.68–1.76)]

BMI, body mass index.

HRs values in bold show statistical significance. HRs adjusted for age, area, smoking (pack-years[PY]=0, 0<PY≤20, >20, or unknown), drinking (never, <1 week/day, current <23 g/day, 23 to <46 g/day, 46 to <69 g/day, ≥69 g/day, or unknown), and history of diabetes (yes, no, or unknown).

† HRs excluding early cases

eTable 5. Distribution of BMI during early adulthood in each category of BMI change among males

**Categories of BMI during early adulthood (kg/m²)**		**Categories of BMI change (BMI at baseline - BMI during early adulthood, (kg/m²))**				
	ALL	**<-4**	**-4 to <-2**	**-2 to <2**	**2 to <5**	**≥5**
**<19**	7,339 (8.1%)	1,045 (12.3%)	659 (5.0%)	2,550 (5.8%)	1,848 (9.8%)	1,237 (22.0%)
**19 to <21**	24,334 (27.0%)	2,362 (27.9%)	3,033 (23.0%)	11,455 (26.0%)	5,627 (29.9%)	1,857 (33.0%)
**21 to <23**	30,249 (33.6%)	2,346 (27.7%)	4,655 (35.3%)	16,363 (37.1%)	5,581 (29.7%)	1,304 (23.2%)
**23 to <25**	18,606 (20.6%)	1,469 (17.3%)	3,227 (24.5%)	9,942 (22.6%)	3,397 (18.1%)	571 (10.2%)
**25 to <27**	6,362 (7.1%)	684 (8.1%)	1,122 (8.5%)	2,809 (6.4%)	1,465 (7.8%)	282 (5.0%)
**27 to <30**	2,649 (2.9%)	426 (5.0%)	407 (3.1%)	826 (1.9%)	723 (3.8%)	267 (4.7%)
**≥30**	584 (0.6%)	135 (1.6%)	78 (0.6%)	107 (0.2%)	158 (0.8%)	106 (1.9%)
**ALL**	90,123 (100%)	8,467 (100%)	13,181 (100%)	44,052 (100%)	18,799 (100%)	5,624 (100%)

BMI, body mass index.

eTable 6. Distribution of BMI during early adulthood in each category of BMI change among males

**Categories of BMI during early adulthood (kg/m²)**		**Categories of BMI change (BMI at baseline - BMI during early adulthood, (kg/m²))**				
	ALL	**<-4**	**-4 to <-2**	**-2 to <2**	**2 to <5**	**≥5**
**<19**	36	7	5	15	7	2
**19 to <21**	107	6	6	61	29	5
**21 to <23**	166	7	31	97	27	4
**23 to <25**	119	9	27	59	23	1
**25 to <27**	47	5	13	18	10	1
**27 to <30**	22	6	3	4	7	2
**≥30**	4	1	3	0	0	0

BMI, body mass index.

eTable 7. Hazard ratios (HR) and 95% confidence intervals (CI) for pancreatic cancer risk according to category of BMI change among females

	**Categories of BMI change (BMI at baseline - BMI during early adulthood, (kg/m^2))**					**Trend (per 1 kg/m^2)**	**Trend *P***	**Heterogeneity [*P*, I^2 (%)]**	
	**<-2**	**-2 to <2**	**2 to <5**	**5 to <8**	**≥8**			**For trend**	**For the highest category**
**Number of subjects (*N*)**	30,233	39,762	23,748	6,897	1,753				
**Person-years (*N*)**	388,829.9	544,533.6	327,601.4	95,363.8	24,671.7				
**Number of cases (*N*)**	126	141	95	30	7				
**Crude rate (per 100,000)**	32.40	25.89	29.00	31.46	28.37				
**HR (model 1) overall**	0.99 (0.77–1.27)	Reference	1.07 (0.73–1.57)	1.24 (0.75–2.05)	1.51 (0.32–7.07)	1.02 (0.97–1.08)	0.483	*P* = 0.007, 71.4	*P* = 0.076, 68.2
**HR (model 2) overall**	1.01 (0.78–1.30)	Reference	1.04 (0.71–1.54)	1.28 (0.79–2.09)	1.56 (0.38–6.39)	1.02 (0.97–1.07)	0.460	*P* = 0.012, 68.8	*P* = 0.108, 61.3
**HR (model 2)† overall**	0.84 (0.63–1.12)	Reference	1.23 (0.90–1.70)	1.31 (0.88–1.94)	1.41 (0.64–3.10)	1.04 (1.00–1.08)	0.080	*P* =0.190, 34.7	*P* = 0.584, 0
**HR (model 1) excluding early cases**	0.96 (0.70–1.31)	Reference	1.12 (0.76–1.64)	1.33 (0.78–2.29)	1.73 (0.40–7.47)	1.03 (0.98–1.09)	0.295	*P* =0.012, 69.1	*P* = 0.096, 63.8
**HR (model 2) excluding early cases**	0.99 (0.75–1.30)	Reference	1.10 (0.74–1.62)	1.40 (0.82–2.39)	1.81 (0.47–6.97)	1.03 (0.98–1.09)	0.272	*P* = 0.017, 67.0	*P* = 0.126, 57.3
**HR (model 2)† excluding early cases**	0.85 (0.62–1.15)	Reference	1.25 (0.86–1.84)	1.46 (0.97–2.21)	1.64 (0.74–3.63)	**1.05 (1.00–1.09)**	**0.049**	*P* = 0.175, 36.9	*P* = 0.673, 0

† HRs were additionally adjusted for BMI during early adulthood (continuous).

Model 1 adjusted for age and area (within each study for JPHC and JACC). Model 2 adjusted for age, area, smoking (pack-years[PY]=0, 0<PY≤20, >20, or unknown), drinking (never, <1 week/day, current <23 g/day, ≥23 g/day, or unknown), and history of diabetes (yes, no, or unknown). HRs values in bold show statistical significance. BMI during early adulthood was evaluated only in JPHC II, JACC, Takayama, Miyazaki, and Ohsaki.

eTable 8. Hazard ratios (HR) and 95% confidence intervals (CI) for pancreatic cancer risk according to BMI during early adulthood stratified by BMI at baseline among females

	**BMI at baseline (kg/m²)**					
	**<21**		**21 to <25**		**≥25**	
**BMI during early adulthood (kg/m²)**	**Cases / Subjects (Person-years)**	**HR (95% CI) [HR (95%CI]†**	**Cases / Subjects (Person-years)**	**HR (95% CI) [HR (95%CI]†**	**Cases / Subjects (Person-years)**	**HR (95% CI) [HR (95%CI]†**
**<21**	35 / 14,431 (195,809.99)	0.70 (0.46–1.07) [0.74 (0.41–1.33)]	79 / 21,505 (295,885.72)	1.19 (0.76–1.86) [1.25 (0.72–2.18)]	22 / 6,643 (91,259.96)	0.98 (0.45–2.17) [1.06 (0.41–2.76)]
**21 to <25**	51 / 9,797 (127,095.47)	1.27 (0.79–2.04) [1.37 (0.80–2.34)]	93 / 23,943 (327,073.11)	Reference	67 / 14,536 (198,447.22)	1.19 (0.69–2.04) [1.21 (0.56–2.59)]
**≥25**	2 / 1,005 (11,225.66)	1.05 (0.24–4.51) [0.71 (0.09–5.44)]	25 / 5,538 (70,223.40)	0.95 (0.59–1.53) [0.98 (0.60–1.59)]	24 / 4,766 (61,888.99)	1.18 (0.74–1.90) [1.33 (0.81–2.19)]

BMI, body mass index.
HRs adjusted for age, area, smoking (pack-years[PY]=0, 0<PY≤20, >20, or unknown), drinking (never, <1 week/day, current <23 g/day, ≥23 g/day, or unknown), and history of diabetes (yes, no, or unknown). HRs values in bold show statistical significance.
† HRs excluding early cases
